# Prior exercise training and experimental myocardial infarction: A systematic review and meta-analysis

**DOI:** 10.6061/clinics/2020/e1293

**Published:** 2020-01-14

**Authors:** Eduardo Carvalho de Arruda Veiga, Brunno Lemes de Melo, Stella de Souza Vieira, Ricardo S. Simões, Vitor E. Valenti, Marcelo Ferraz Campos, Joseane Elza Tonussi Mendez Rossetti do Vale, Roberta Lukesvicius Rica, José Maria Soares-Júnior, Edmund Chada Baracat, Andrey Jorge Serra, Julien S. Baker, Danilo Sales Bocalini

**Affiliations:** I Disciplina de Ginecologia, Departamento de Ginecologia e Obstetricia, Hospital das Clinicas HCFMUSP, Faculdade de Medicina, Universidade de Sao Paulo, Sao Paulo, SP, BR; II Divisao de Cardiologia, Departamento de Medicina, Universidade Federal de Sao Paulo, Sao Paulo, SP, BR; III Programa de Pos-Graduacao em Fisioterapia, Universidade Estadual de Sao Paulo (UNESP), Presidente Prudente, SP, BR; IV Disciplina de Delineamento de Estudos e Escrita Cientifica, Centro Universitario Saude ABC, Santo Andre, SP, BR; V Secretaria de Estado da Saude do Acre, Acre, AC, BR; VI Departamento de Educacao Fisica, Universidade Estacio de Sa, Vitoria, ES, BR; VII Institute for Clinical Exercise and Health Sciences, School of Health and Life Sciences, the University of the West of Scotland, Lanarkshire, Scotland; VIII Department of Sport and Physical Education, Faculty of Social Sciences, Centre for Health and Exercise Science Research, Hong Kong Baptist University, Kowloon Tong, Hong Kong, China; IX Laboratorio de Fisiologia e Bioquimica Experimental, Centro de Educacao Fisica e Deportos, Universidade Federal do Espirito Santo, Vitoria, ES, BR

**Keywords:** Prior Exercise, Experimental Myocardial Infarctions, Systematic Reviews, Meta-Analysis, Exercise Training, Swimming, Running

## Abstract

Exercising prior to experimental infarction may have beneficial effects on the heart. The objective of this study was to analyze studies on animals that had exercised prior to myocardial infarction and to examine any benefits through a systematic review and meta-analysis. The databases MEDLINE, Google Scholar, and Cochrane were consulted. We analyzed articles published between January 1978 and November 2018. From a total of 858 articles, 13 manuscripts were selected in this review. When animals exercised before experimental infarction, there was a reduction in mortality, a reduction in infarct size, improvements in cardiac function, and a better molecular balance between genes and proteins that exhibit cardiac protective effects. Analyzing heart weight/body weight, we observed the following results - Mean difference 95% CI - -0.02 [-0.61,0.57]. Meta-analysis of the infarct size (% of the left ventricle) revealed a statistically significant decrease in the size of the infarction in animals that exercised before myocardial infarction, in comparison with the sedentary animals -5.05 [-7.68, -2.40]. Analysis of the ejection fraction, measured by echo (%), revealed that animals that exercised before myocardial infarction exhibited higher and statistically significant measures, compared with sedentary animals 8.77 [3.87,13.66]. We conclude that exercise performed prior to experimental myocardial infarction confers cardiac benefits to animals.

## INTRODUCTION

Meta-analyses and guidelines concluded that the engagement in exercise training (ET) is known to have many health benefits, including prevention for all-cause mortality, reduced risk of ischemic heart disease, and mortality (1,2). One additional role of ET extends to its possible action in protecting the heart against myocardial infarction (MI). This issue is interesting because approaches to prevention of heart disease may fail, and future projections have addressed increases in MI and heart failure for decades to come (3,4). Unfortunately, little literature has appeared addressing the influence of ET before a MI and previous observational studies have reported unclear findings. A cross-sectional study showed that veteran athletes with lifelong ET were related to attenuated pathological cardiac remodeling after MI, as illustrated by a superior left ventricular (LV) ejection fraction and LV circumferential strain compared to infarcted-sedentary participants (5). A prospective study with 1,517 MI events reported that participants who engaged in light or moderate physical activity were more likely to survive to MI (6). On the other hand, in a study with 504 patients with acute coronary syndrome, activity levels did not affect the severity of the disease assessed by The thrombolysis in myocardial infarction (TIMI) risk score (7).

Animal studies have shown that ET prior to MI can attenuate pathological LV remodeling (8-20). Experimental studies make it possible to directly assess the impact of ET on the infarcted heart while clinical studies have categorized the physical activity level through subjective assessment using questionnaires to determine prognosis after MI. Furthermore, more specific markers of cardiac remodeling (e.g., cardiomyocyte hypertrophy, cell death, and molecular tissue signaling) can be readily assessed under experimental conditions.

The first study that evaluated the role of ET prior to MI was published over four decades ago (8), and research indicates that increased myocardial capillary density in rats led to permanent coronary occlusion. Other benefits include LV performance, apoptosis, fibrosis, and myocardial inflammation as well as mortality in infarcted rodents (12,14,15), the potential protective of ET remains incompletely understood. For example, some studies reported no benefit of prior ET in terms of attenuating myocardial hypertrophy, LV systolic and diastolic dysfunction, and inotropic myocardial depression in infarcted rats (14,15). Moreover, the small number of studies evaluating how ET prior to MI affects cardiac remodeling considered distinct experimental designs, type (e.g. treadmill or swimming), and exercise regimens (18-23). Therefore, the difference study findings must be understood better in an integrated manner in order to confirm whether ET protects against maladaptive remodeling post-MI.

Therefore, we conducted a meta-analysis to examine the association between ET prior to MI and cardiac remodeling in experimental models. Specifically, we examined the effects of ET on LV structure and function, myocardial growth, cell death, and gene/protein expression.

## MATERIAL AND METHODS

**Figure 1:**
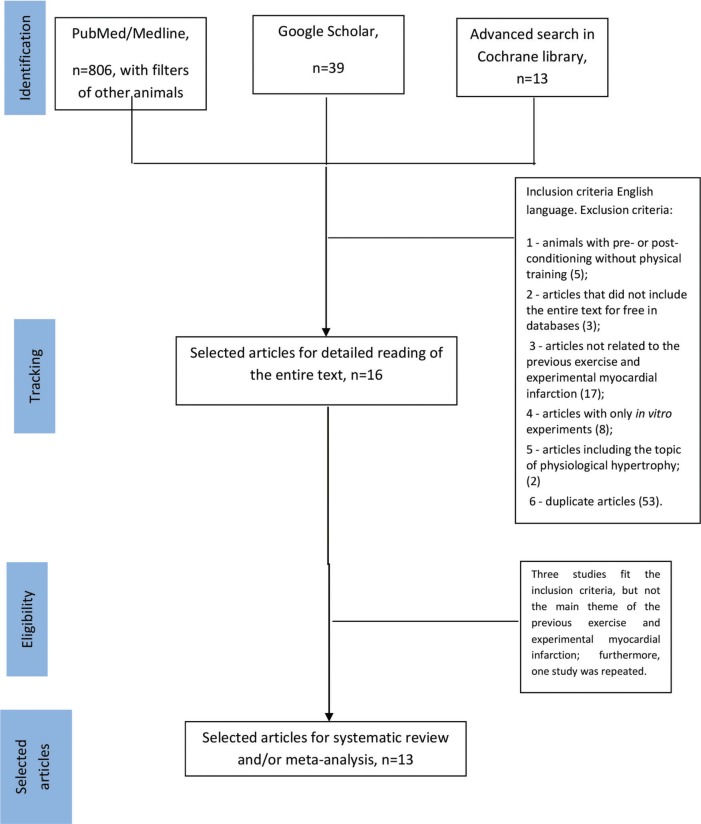
Flowchart for selection of studies.

This search strategy was completed by following with Yoshii et al. (24). For the identification of the studies included or considered in this review, articles from January 1978 to November 2018: in PUBMED, Google Scholar, and Cochrane were considered. First, we selected keywords from related articles and MeSH international data lines were used to find more related key words with close meanings, these included: (“prior exercise training” [MeSH Terms] OR (“exercise” [All Fields] AND (“myocardial infarction”) [MsSH Terms] [All Filds] OR (“physical exercise” [MeSH Terms] “physical examination” OR “exercise training” [MeSH Terms] “exercise” AND “myocardial infarction”. The search strategy was carried out on three databases. PubMed searches including the other animal filters resulted in potentially 806 articles. Google Scholar with the filter only in the title resulted in 39 articles and the Cochrane database which resulted in 13 articles all in humans. The terms used were “prior exercise training and myocardial infarction” MeSH terms: myocardial infarction; exercise; the search was repeated following review of the eligible papers to specifically search for methodologies, outcomes, and parameters of myocardial infarction. We also reviewed the retrieved articles to identify possible additional studies (Figure 1). This review was conducted according to the recommendations established by PRISMA (Preferred Reporting Items for Systematic Reviews and Meta-Analysis) (25).

The present study did not include studies with ischemia and reperfusion or ischemic preconditioning and those assessing only animals that underwent experimental myocardial infarction surgery with mechanical occlusion of the left coronary artery. The control group was the sham group, (the same procedures used for experimental surgery for myocardial infarction was followed, but without occlusion of the left coronary artery). Most of the work was performed using male rats and only one study used a transgenic mouse.

The process of retrieving the papers, as well as evaluating the titles and abstracts obtained, was conducted by two researchers with the ability to perform systematic reviews (E.C.V. and V.E.V.) independently and blindly, following the inclusion and exclusion criteria according to the components of P.I.C.O. (22). The selected articles were then critically evaluated to be included or excluded in the review. If the reviewers disagreed on the selection of a study, a third reviewer was consulted (S.S.V.).

The information obtained from the studies selected for the systematic review are presented in a table where the following characteristics of the articles were described: authors name and year of publication, animal type, sex (M/F), animal race, age (months), weight, induction model, and ethical committees (Table 1). The RevMan version 5.3 (Cochrane Collaboration, Oxford, UK) was used to perform the meta-analysis. The random-effect model was used in the period of heterogeneity.

### Statistical Analysis

The mean values and standard deviation between the studies are presented as mean difference (MD) of the post-intervention values after calculating the inverse of the variance, which were used to determine the magnitude of the protective effect of exercise prior to myocardial infarction experimental surgery (26). Heterogeneity was assessed through Cochran and I2 Q tests, followed by visual inspection of the graph.

The heterogeneity between the studies was analyzed through the I2 statistic (27). The analyses were performed using Program R 3.3.1 for Macintosh.

## RESULTS

The search process, identification, and selection of the articles are shown in Figure 1. Of the search strategies used, 806 articles in PUBMED with the filters of other animals were found, as a result 38 articles were retrieved, of which 16 were selected after reading the title and abstract; in the search of google scholar we obtained 39 articles and in the search by Cochrane database we obtained 13 articles. The inclusion and exclusion criteria are described in figure 1.

Besides the fact that they were not related to the components of P.I.C.O., most of the articles were excluded because they involved human, rather than animal, subjects. Table 1 presents the data on authors, animal type, sex, animal race, age (months), weight, and induction model of experimental myocardial infarction of artery coronary occlusion (28,29). Table 2 presents the data on authors, sample size, number of groups, number of animal/groups, the methodology of exercise, functional fitness, and time for the sacrifice of the animal after the beginning of the physical training, dependent variables.

Table 3 shows the study characteristics of selected experimental controlled animal studies with prior exercise and myocardial infarction being divided into Positive effects that were statistically significant. Concerning to measures of infarct size, these authors found a statistical difference between the animals that were previously exercised and the sedentary ones (8,11-13,16), while other authors did not find statistical significance (14,15,17-19). Measures related to hypertrophy as heart weight/body weight were found to be statistically different from the following authors (9,10,13), while in other studies the variables were not significant (9,12,14,15). Several methods were used for the measurement of capillary density and all the authors who performed this measure found statistical significance (8,10,12,13,20).

Outlined in Table 3, the following studies reported a Statistical difference between heart function variables such as in the variable ejection fraction [with the equivalent measure in animals on the echocardiogram the fraction of shortening the transverse area expressed as a percentage of the amount of blood ejected from the left ventricle] (9-11,13,20); however, other studies reported no significant difference (14,15,18). In the hemodynamic measures, significance was found in the following studies (12,16,19). The remaining collagen content in the remaining myocardium was lower in the animals that were exercised in the following studies (12,16). The rate of apoptosis in the remaining myocardium of the exercised animals was lower, with statistical significance in these studies (12,18,20).

When the molecular profile of the exercised animals was analyzed concerning the sedentary ones, each study analyzed different genes or proteins with statistical significance (Table 3). The first work to analyze the genes that gained the most benefit from exercise was that of Freimann et al. (10) who obtained excellent results using the microarray technique. The author highlighted the following genes that had greater expression: ANP, COX 6A, COX 8h, H-FABP, and Aldalose. Freimann et al. (11) highlighted the following analyzed genes that obtained expressive improvements with exercise. These included the genes from ANP, collagen I and III, and PCPE. Tang et al. (13) also analyzed the molecular profile highlighting the increased expression of the VEGF protein. Santos et al. (18) analyzed the inflammatory profile of the proteins and verified an important result with the expressions of the proteins NF-_Κ_B, PPAR-α and TNF-α with better expressions in exercised and infarcted animals. Barbosa et al. (19) also found an improvement in the inflammatory profile in animals with previous exercise and infarction in the following cytokines TNF-α, IL6, and IL10. Vieira et al. (20) showed significant improvement by analyzing the following proteins pAkt1 and VEGF.

A meta-analysis of the protective effect of exercise prior to the experimental surgery of myocardial infarction.

**Figure 2:**
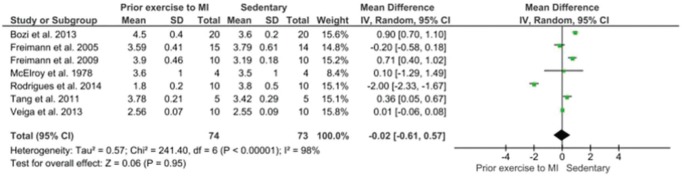
Meta-analysis of body weight/heart weight (mg/g). Forest Plot of heart weight compared to prior exercise and sedentary; confidence interval (CI); the standardized mean difference (SMD), standard deviation (SD).

Analyzing heart weight/body weight, we observed the following results - Mean difference 95% CI - -0.02[-0.61,0.57] (Figure 2).

**Figure 3:**
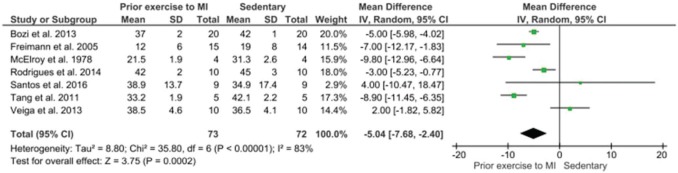
Meta-analysis of infarcted size measurement by echocardiography (% left ventricular). Forest Plot of myocardial infarction size compared to prior exercise and sedentary; confidence interval (CI); the standardized mean difference (SMD), standard deviation (SD).

Following the meta-analysis of the infarct size (% LV), it was observed that the animals that were involved in prior exercise before myocardial infarction had a decrease in infarct size, statistically significant, in comparison with the sedentary ones -5.05[-7.68, -2.40] (Figure 3).

**Figure 4:**
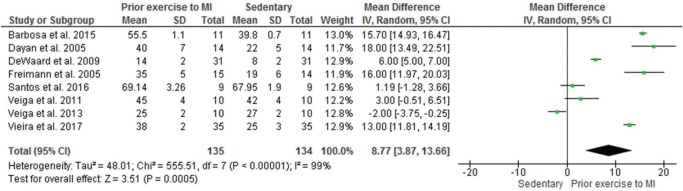
Meta-analysis of ejection fraction measured by echocardiography (% left ventricular). Forest Plot of ejection fraction compared to prior exercise and sedentary; confidence interval (CI); the standardized mean difference (SMD), standard deviation (SD).

When we analyzed the ejection fraction measured by echo (%LV), it was verified that animals with prior exercise had higher and statistically significant measures than the sedentary ones 8.77[3.87, 13.66] (Figure 4).

The meta-analysis aimed to investigate the magnitude of the protective effect of exercise prior to the experimental surgery of myocardial infarction on the variables of left ventricular diameter in the diastole and ejection fraction of exercise and infarction.

## DISCUSSION

Our results suggest that exercise performed before experimental myocardial infarction improves survival and cardiac function as well as the molecular response of gene and proteins involved in heart failure. In general, exercise determined a decrease in the mortality and inflammation rates related to ventricular remodeling, extracellular matrix and apoptosis processes, including cell signaling and other cellular activities. In addition, those mechanisms result in better ejection fraction of animals under exercise before the myocardial infarction.

An important aspect of the studies analyzed with exercise prior to experimental myocardial infarction is the ability to determine the size of the infarct in the heart of rats (10,12). While others had no effect on the reduction of infarct size, but had other beneficial consequences for the heart (17-20), others had zero responses to the heart benefits from the previous exercise (8,9). Another important aspect is that in some studies, mortality decreased (12,17-20). It is crucial to emphasize that the extension of the infarcted area may impact on heart function (12-20). Therefore, this reduction reduces the mortality as well as the sequels that may generate dysfunction and heart failure (12-20). Our meta-analysis indicates that exercise improves heart function (Figure 4), which reflects the strength of hearing muscles and the coordination of the heart system. This is a great benefit of exercise. The differences that occurred in the meta-analysis between the size of the myocardial scar (8,10,13,16,17) were significantly different in ejection fraction (9,10,12,17,18), whereas others (15,19) did not show statistically significant changes in ejection fraction.

Another benefit of exercise was to increase the response of stress-related proteins and aerobic metabolism that protect the heart. In addition, the inflammation, pro-apoptosis, and the fibrotic process of animals under exercise presented a better profile compared to sedentary ones (12-20). In addition, the genes and proteins involved in the repair process and heart protection show a greater expression in those who underwent previous exercise: ANP, COX 6A, COX 8 h, H-FABP, Aldalose, collagen I and III and PPAR-α (10,11,18). Some studies did not find a significant change in the echocardiographic variable rats under exercise program, which is another important factor (8,11).

Rodrigues et al. (11) conducted a study in which infarcted rats underwent treadmill training which reduced mortality and improved the molecular inflammatory system. De Waard et al. (12) also noted that physical exercise in mice submitted to experimental infarction increased survival. In this context, treadmill training in rats caused drop-in mortality in the infarcted group and improved the inflammatory and apoptosis systems (18). The decrease in mortality and inflammatory resulting from physical exercise was verified by Barbosa et al. (19). The inflammatory response may increase heart damage after ischemia. Therefore, exercise may determine a positive effect in the heart through a reduction in the inflammatory process.

The heart weight/body weight data showed a significant gain in heart mass after exercise which is indicative of physiological hypertrophy. However, the study by McElroy et al. (8) only included four animals per group resulting in a large confidence interval in the analysis that did not allow the results to reach significance. In the meta-analysis of the comparison of infarct size, measured by an echocardiogram (% of the left ventricle), it was observed that animals that exercised before infarction had a significant decrease in infarct size. When the ejection fraction was analyzed, we also observed an increase in blood ejected from the left ventricle in animals that had prior exercise training was statistically significant.

The current scenario in human literature that assesses exercise prior to infarction relates to the increased survival, including the recommendations of the current prevention guidelines regarding the main effects of exercise already observed at low to moderate levels (30). According to Ejlersen et al. (30), humans with moderate to high levels of leisure-time physical activity, reduce the risk of a fatal post-infarction event by 45%, corroborating the findings of this study; however, the cardioprotective effects were not found in the development of heart failure. In a study accompanying more than 6,000 patients, Gonzales et al. (31), describes the lower incidence of myocardial infarction event in individuals exposed to physical exercise, also suggesting that the role of physical exercise is more important for men than for women throughout the study. In a prospective study with the Chinese population, it was found that an increase in physical activity level (which could be obtained from occupational or non-occupational activities) was associated with a statistically significant 5% to 12% lower risk of different subtypes of cardiovascular disease, including myocardial infarction (32). Kupsky et al. (33), whose report describes exercise-derived cardioprotection in a cohort study of more than 60,000 subjects analyzed, also strengthen these data.

In conclusion, most studies showed that exercise performed before the infarction triggers several benefits. The major benefits include a reduction in mortality, improvement of the ejection fraction, and an increase in the molecular profile, which may provide a more favorable adaptation to remodeling.

## AUTHOR CONTRIBUTIONS

Veiga ECA provided substantial contributions to the concept and design of the study, and definition of intellectual content, was involved in literature search, data analysis, statistical analysis, manuscript preparation, drafting, writing, critical review for important intellectual content, and approval of the final manuscript version to be published. Melo BL was involved in literature search, data analysis, statistical analysis, manuscript preparation, writing, drafting and critical review for important intellectual content, and approval of the final manuscript version to be published. Vieira SS, Valenti VE, Simões RS, Campos MF, Vale JETMR and Rica RL were involved in literature search, data analysis, statistical analysis, and approval of the final version of the manuscript to be published. Soares-Junior JM, Baracat EC, Serra AJ, Baker JS and Bocalini DS provided substantial contributions to the concept and design of the study, and definition of intellectual content, manuscript writing, drafting or critical review for important intellectual content, and approval of the final manuscript version to be published.

## Figures and Tables

**Table 1:** Characteristics of selected control experiment studies of prior exercise and experimental myocardial infarction.

Authors	Animal	Sex	Animal race	Age (months)	Weight (g)	Induction model
McElroy et al. (8)	Rats	Male	Sprague-Dawley	1	-	MI
Dayan et al. (9)	Rats	Male	Sprague-Dawley	-	-	MI
Freimann et al. (10)	Rats	Male	Sprague-Dawley	-	250-290	MI
de Waard et al. (12)	Mice	Either sex	Wild type C57B1/6	-	-	MI
Freimann et al. (11)	Rats	Male	Sprague-Dawley	-	250-290	MI
Tang et al. (13)	Rats	Male	Sprague-Dawley	-	245-289	MI
Veiga et al. (14)	Rats	Female	Wistar	3	250-300	MI
Bozi et al. (16)	Rats	Male	Wistar	-	100-120	MI
Veiga et al. (15)	Rats	Female	Wistar	2	190-200	MI
Rodrigues et al. (17)	Rats	Male	Wistar	3	275-300	MI
Santos et al. (18)	Rats	Female	Wistar	3	250-290	MI
Barbosa et al. (19)	Rats	Male	Wistar	3	250-300	MI
De Souza Vieira et al. (20)	Rats	Female	Fisher 344	-	140-190	MI

Experimental myocardial infarction (MI) technique by coronary occlusion consolidated by the literature (Wu et al. (28)).

**Table 2:** Study of the characteristics (samples size, number of groups, number of animals / group, dependent variables) of selected experimental studies of controlled animals on effects of prior exercise and experimental myocardial infarction.

Authors	Sample size	Number of groups	Number of animals /groups	Methodology of exercise	Funcional fitness	Exercise intensity	Time of Training and detraining	Dependent variables
McElroy et al. (8)	8	2	4	Swimming 1h/day, 5 days/week, for 5 weeks	-	-	48 hours of T- 2 days of DET	IF, biometrics analysis, determination of capillary/fiber ratio.
Dayan et al. (9)	35	3	14 (Ex), 14 (Sed), 7 (Control)	Swimming 90 min/day, 5 days/week for 3 weeks	-	60-65% of VO2 máx	3 weeks of T - 4 weeks of DET.	Heart characteristics, Echo.
Freimann et al. (10)	-	-	-	Swimming 90 min/day, 5 days/week for 7 weeks	-	60-65% of VO2 máx	11 weeks of T - 3 weeks of DET.	IF, blood vessels count, echo, analysis of gene expression.
de Waard et al. (12)	186	6	-	2 weeks of voluntary wheel running	30% increase of skeletal muscle citrate synthase activity	60-65% of VO2 máx	16 weeks of T - 8 weeks of DET.	Survival, IF, determination of capillary/fiber ratio, capillary density, collagen content, apoptosis.
Freimann et al. (11)	-	-	-	Swimming 90 min/day, 6 days/week for 7 weeks	-	60-65% of VO2 máx	4 hours, 2 days and 4 weeks of T -	DNA microarray analysis, RT-q PCR, heart and body weight, gene expression profiles.
Tang et al. (13)	30	6	5	Treadmill running exercise 60 min/day, 5 days/week for 6 weeks	Levels of plasma lactic acid examined	High-intensity; moderate intensity; low intensity	7 weeks of T- one week of DET.	IF, determination of capillary/fiber ratio, micro vessel density, western blot analysis of VEGF.
Veiga et al. (14)	44	4	11	Swimming 60 min/day, 5 days/week for 8 weeks	Physical capacity swin test (ref)	60-65% of VO2 máx	9 weeks of T- one week of DET.	Evaluation of area of risk of infarcted myocardial, Echo.
Bozi et al. (16)	55	3	15 (Sham), 20 (SedMI), 20 (EXMI)	Aerobic exercise training running 60 min/day, 5 days/week for 8 weeks	Underwent a graded treadmill exercise training until exhaustion for 3 days with 2 different observers	55-70% of exercise intensity	10 weeks of T- 2 weeks of DET.	Determination of capillary/fiber ratio, in situ LV pressure-volume relationship, histologic evaluation, cardiomyocyte contractile function and morphology.
Veiga et al. (15)	40	4	10	Swimming 60 min/day, 5 days/week for 8 weeks	Physical capacity swin test (ref)	60-65% of VO2 máx	12 weeks of T- 4 weeks of DET.	IF, determination of capillary/fiber ratio, papillary measurement.
Rodrigues et al. (17)	40	4	10	Treadmill running exercise 60 min/day, 5 days/week for 8 weeks	Maximal running speed achieved in the test present a good correlation with the maximum oxygen consumption	72 to 75% of VO2 max	8.5 weeks of T- few days of DET.	IF, determination of capillary/fiber ratio, LV morphometry and function, cardiac autonomic modulation.
Santos et al. (18)	54	6	9	Swimming 60 min/day, 5 days/week for 8 weeks	-	60-65% of VO2 máx	12 or 9 weeks of T- 1 or 4 weeks of DET.	IF, echo, immunohistochemistry, antigen quantification, apoptosis.
Barbosa et al. (19)	42	4	11	Treadmill running exercise 60 min/day, 5 days/week for 8 weeks	-	Low to moderate intensity	8.5 weeks of T - few days of DET.	Determination of capillary/fiber ratio, cardiovascular autonomic modulation, cytokines concentration by ELISA.
Vieira et al. (20)	130	4	35	Swimming 90 min/day, 5 days/week for 9 weeks	VO_2_ peak (ml.kg^-1^.min^-1^) was used as marked of cardiorespiratory fitness	3x greater exercise intensity	13 weeks of T -4 weeks of DET	IF, echo, papillary measurements, apoptosis assay, histomorphometry, immunoblotting

T - training; DET - detraining; IF - measurements of infarcted size by an echocardiogram (% left ventricle); Echo - echocardiography measurements; CF - cardiac function was determinate as echocardiography and hemodynamics measurements. Moderate intensity is based on this reference (Bocalini et al. (34)) that says that the rats had swimming around 60% of VO2max.

**Table 3:** Study characteristics of selected experimental controlled animal study of positive effects- statistically significant among the measures evaluated.

Authors	Measurements of the infarcted size	HW/BW	Capillary density	Ejection fraction	Hemodynamic measurements	Collagen	Apoptosis	Benefits of gene/protein expression with prior exercise
McElroy et al. (8)	X	NS	X	-	-	-	-	-
Dayan et al. (9)	-	X		X	-	-	-	-
Freimann et al. (10)	X	X	X	X	-	-	-	X
de Waard et al. (12)	X	NS	X	X	X	X	X	
Freimann et al. (11)	-	X	-	-	-	-	-	X
Tang et al. (13)	X	X	X	X	-	-	-	X
Veiga et al. (14)	NS	NS	-	NS	-	-	-	-
Bozi et al. (16)	X	X	-	-	X	X	-	-
Veiga et al. (15)	NS	NS	-	NS	NS	-	-	-
Rodrigues et al. (17)	NS	-	-	X	NS	-	-	-
Santos et al. (18)	NS	-	-	NS	-	-	X	X
Barbosa et al. (19)	NS	-	-	X	X	-	-	X
Vieira et al. (20)	NS	-	X	X	-	-	X	X

X - the difference of the variable between the sedentary animal before the experimental myocardial infarction surgery and the animal with the previous exercise before the experimental myocardial infarction surgery was statistically significant. NS - not significant. - (-) means that in this work this specific variable was not used.
